# Attempting to counteract vigilance decrement in older adults with brain stimulation

**DOI:** 10.3389/fnrgo.2023.1201702

**Published:** 2023-12-12

**Authors:** Birte S. Löffler, Heiko I. Stecher, Arnd Meiser, Sebastian Fudickar, Andreas Hein, Christoph S. Herrmann

**Affiliations:** ^1^Assistance Systems and Medical Device Technology, Department of Health Services Research, Carl von Ossietzky University Oldenburg, Oldenburg, Germany; ^2^Experimental Psychology Lab, Department of Psychology, European Medical School, Cluster of Excellence “Hearing4all”, Carl von Ossietzky University Oldenburg, Oldenburg, Germany; ^3^Research Center Neurosensory Science, Carl von Ossietzky University Oldenburg, Oldenburg, Germany

**Keywords:** alpha power, brain stimulation, EEG, enhancement, gamma-tACS, older adults, reaction times, vigilance

## Abstract

**Introduction:**

Against the background of demographic change and the need for enhancement techniques for an aging society, we set out to repeat a study that utilized 40-Hz transcranial alternating current stimulation (tACS) to counteract the slowdown of reaction times in a vigilance experiment but with participants aged 65 years and older. On an oscillatory level, vigilance decrement is linked to rising occipital alpha power, which has been shown to be downregulated using gamma-tACS.

**Method:**

We applied tACS on the visual cortex and compared reaction times, error rates, and alpha power of a group stimulated with 40 Hz to a sham and a 5-Hz-stimulated control group. All groups executed two 30-min-long blocks of a visual task and were stimulated according to group in the second block. We hypothesized that the expected increase in reaction times and alpha power would be reduced in the 40-Hz group compared to the control groups in the second block (INTERVENTION).

**Results:**

Statistical analysis with linear mixed models showed that reaction times increased significantly over time in the first block (BASELINE) with approximately 3 ms/min for the SHAM and 2 ms/min for the 5-Hz and 40-Hz groups, with no difference between the groups. The increase was less pronounced in the INTERVENTION block (1 ms/min for SHAM and 5-Hz groups, 3 ms/min for the 40-Hz group). Differences among groups in the INTERVENTION block were not significant if the 5-Hz or the 40-Hz group was used as the base group for the linear mixed model. Statistical analysis with a generalized linear mixed model showed that alpha power was significantly higher after the experiment (1.37 μV^2^) compared to before (1 μV^2^). No influence of stimulation (40 Hz, 5 Hz, or sham) could be detected.

**Discussion:**

Although the literature has shown that tACS offers potential for older adults, our results indicate that findings from general studies cannot simply be transferred to an old-aged group. We suggest adjusting stimulation parameters to the neurophysiological features expected in this group. Next to heterogeneity and cognitive fitness, the influence of motivation and medication should be considered.

## 1 Introduction

In 2018, we found that the application of transcranial alternating current stimulation (tACS) on the visual cortex significantly reduced the slowdown of reaction times in a vigilance experiment in a group of young adults (Löffler et al., 2018). We set out to repeat the study but with older adults.

In Europe, as well as other industrialized countries, the demography is changing toward an older society: By 2070, the number of people aged 65 years and older will mount up to 30% of the total population in Europe (European Commission, 2020). Due to the population aging, more people will likely be required to work longer before retirement and therefore make up a huge portion of not only car and bicycle drivers but also the active workforce. Getting old does not come without costs: In aging, neurobiological, cognitive, and behavioral changes are seen (Hedden and Gabrieli, 2004; Grady, 2012). Aging may have severe effects on the brain and often leads to a continuous and reliable decline in numerous perceptual and cognitive functions (Hedden and Gabrieli, 2004; Salthouse, 2010) with only rare treatment options (Abbott, 2012). These effects can be mainly observed not only in behavioral and neuropsychological tasks assessing speech perception, working memory, processing speed, executive functions, reasoning, and spatial orientation (Hedden and Gabrieli, 2004; Salthouse, 2010) but also in rapid mental fatigue when performing a long-lasting task (Wascher and Getzmann, 2014)—important functions for activities mentioned by older adults as relevant for a good, participative lifestyle (Owsley, 2002).

Vigilance is the capability to stay alert and ready to react to prolonged tasks (Warm et al., 2008) and represents an executive function (Cristofori et al., 2019). Vigilance decrement is the decline in performance with time on task, for example, expressed by rising reaction times and reduced detection rates (Buck, 1966; Pattyn et al., 2008). Vigilance decrement is thought to be responsible for numerous accidents and other safety-critical events. It is becoming more relevant lately due to the propagation of automatization and the increasing number of monitoring tasks (Dinges, 1995). Therefore, much research focuses on investigating vigilance-decrement detecting systems (McWilliams and Ward, 2021; Tamanani et al., 2021) and vigilance-enhancing strategies (see Al-Shargie et al., 2019, for a review).

Conventional vigilance-enhancing strategies include caffeine or chewing gum (Al-Shargie et al., 2019). Recent research has shown that an increase in cognitive load works beneficial: Vigilance is enhanced by integrating challenges to monitoring tasks like artificial rain (Bodala et al., 2016), adding visual and haptic stimuli (Abbasi et al., 2017) or a pure audio tone at 250 Hz (Al-Shargie et al., 2021). Another promising enhancing strategy is transcranial electrical stimulation (TES), which is an easy-to-apply and relatively low-cost technique ideal for private and mobile applications (Antal and Paulus, 2013). In TES, two (or more) electrodes are placed on the scalp that are used to induce low electrical fields. Successful vigilance enhancement has been shown for transcranial direct current stimulation (tDCS; McIntire et al., 2014; Nelson et al., 2014) and tACS (Löffler et al., 2018; Rostami et al., 2021). In tDCS, stimulation electrodes are used as an anode and a cathode, leading to a de- or hyperpolarization of the exposed brain tissue. Negative aspects of this method are the enhancement of cognitive functions at the expense of other cognitive functions (Iuculano and Cohen Kadosh, 2013) or opposing effects depending on the individual's state (Sarkar et al., 2014). tACS uses alternating current and allows direct interference with the ongoing oscillatory brain activity with no serious adverse events reported as of 2017 (Matsumoto and Ugawa, 2017). Applied in the conventional electroencephalogram (EEG) frequency range, tACS is believed to modulate brain oscillations by the synchronization of neuronal networks and has been shown to induce behavioral and neurophysiological effects that occur immediately (on-line) but have also been shown to outlast stimulation (off-line or so-called aftereffects; Kasten et al., 2016). Lately, tACS has gained broad interest as a possible therapeutic method in treating neuropsychological disorders linked to abnormal brain oscillations, such as Parkinson's disease (Guerra et al., 2020) or dementia (Moussavi et al., 2021) and Alzheimer's disease (Sprugnoli et al., 2021). Several studies showed that the application of tACS can induce a behavioral change and increase performance (for a review, see Klink et al., 2020a), making it a promising tool for neuro-enhancement. In the context of vigilance, it has been applied with mixed results. While stimulating the medial prefrontal cortex with 6-Hz tACS improved performance in a visual sustained attention task (Rostami et al., 2021), stimulating with 4 Hz and 10 Hz did not reduce vigilance decrement (van Schouwenburg et al., 2021).

In our study (Löffler et al., 2018), we reduced vigilance decrement by stimulating the visual cortex with 40 Hz. We combined two insights to conceptualize the study. First, we combined neurophysiological knowledge and then, second, looked for the appropriate stimulation setup.

First, rising reaction times and worsening detection rates show a positive correlation with time on task (Buck, 1966)—a concept widely used in vigilance research (for review, see, e.g., Oken et al., 2006; Pattyn et al., 2008). Furthermore, on a neurophysiological scale, reaction time slowing and vigilance decrement have been linked to rising posterior alpha power with time on task (Klimesch, 1999; Schmidt et al., 2009; Molina et al., 2013; Clayton et al., 2015). The rise in posterior alpha power is associated with experienced mental fatigue (Craig et al., 2012) and relates to reaction time slowing (Klimesch et al., 1996). These relationships led us to the conclusion that reaction time slowing can be prevented if alpha power is downregulated. The latter has successfully been demonstrated by Helfrich et al. (2014a, 2016) who down-modulated alpha amplitudes with gamma tACS. They stimulated the visual cortex with 40 Hz and a high-definition tACS electrode montage, where five electrodes were positioned on each hemisphere, allowing stimulation in or with 180° phase difference between them. Notably, downregulation of alpha amplitudes was observed independently of in- or antiphase stimulation.

A possible reason for the down-modulating effect derives from a phenomenon called cross-frequency coupling (CFC), also known for other frequencies: Brain oscillations of specific frequencies interact with each other (Jensen and Colgin, 2007). In general, during CFC, specific subharmonic sets of faster and slower oscillations are nested in each other and modify each other. CFC is believed to be a mechanism for information transfer in nested or coupled neuronal networks and may provide information integration across several spatiotemporal scales (Canolty and Knight, 2010). CFC can occur in different ways, depending on its function and whether the slow or fast oscillation is master or slave (Helfrich et al., 2016). Coupling can occur between amplitudes, power, phase, or frequency (Abubaker et al., 2021). It is believed that amplitude coupling regulates the activation of distributed neuronal populations, while phase coupling mediates specific inter-areal cortical information flow (Engel et al., 2013). In their study, Helfrich et al. (2016) showed that gamma-alpha CFC gamma-band entrainment enhanced amplitude–envelope correlations and reduced alpha power, indicating an antagonistic relationship between them. They concluded that coupled alpha and gamma oscillations have a functional role in visual processing. Also, other studies showed that gamma and alpha frequencies interact during cognitive processes, with the strongest coupling over occipital areas (Palva et al., 2005; Osipova et al., 2008). Therefore, we decided to investigate the possibility of gamma tACS to downregulate the expected rise in occipital alpha power on the visual cortex, this time addressing older adults.

In 2016, the first successful tACS study targeting healthy older adults aged 60 years and older improving implicit language learning skills was published (Antonenko et al., 2016). Since then, most studies have investigated the impact of tACS on the prefrontal cortex (PFC), addressing higher cognitive functions like working memory (Reinhart and Nguyen, 2019; Draaisma et al., 2022), associative memory encoding (Klink et al., 2020b) and multitasking (Zanto et al., 2021). Furthermore, tACS stimulation of the PFC has been shown to support cognitive training in cases of dementia (Moussavi et al., 2021). Other successful tACS studies enhanced motor functions (Guerra et al., 2021) and motion learning (Rumpf et al., 2019; Fresnoza et al., 2020) or addressed auditory functions (Rufener et al., 2016; Baltus et al., 2020). One study found that alpha tACS (but not theta or gamma) at parietal regions improved performance in a working memory paradigm (Borghini et al., 2018). Only a few studies have compared young and old target groups using the same protocol and stimulation setup (Rufener et al., 2016; Reinhart and Nguyen, 2019; Fresnoza et al., 2020; Guerra et al., 2021; Zanto et al., 2021).

We repeated our study (Löffler et al., 2018) but with older adults to analyze the impact of identical tACS setup on different age groups and test if the setup is beneficial for older adults as well. In order to validate our hypothesis of a vigilance-induced rise of alpha power and its downregulation by gamma tACS, we extended the study by recording EEG and an additional 5-Hz control group to ensure that any enhancing effects are frequency-specific (Davis et al., 2013). We used the same study design consisting of two blocks—a baseline and an intervention—and recorded the EEG before and after both blocks. We expected that our experiment would lead to a vigilance decrement expressed by rising reaction times over time on task in the baseline block and higher alpha power post the experiment for the sham and control group. We hypothesized that our intervention with 40-Hz tACS would lead to a significantly flatter slope of increased reaction times in the intervention block and a significantly lower level of alpha power after the experiment as compared to the sham and 5-Hz control groups.

To the best of our knowledge, no study has previously applied 40-Hz gamma tACS on the visual cortex to address vigilance decrement in older adults. We believe that this research has high technical relevance given the changing demographics and increasing number of monitoring tasks such as automated driving (Gartenberg et al., 2018), which older adults cite as important for participating in society and as part of a good quality of life (Owsley, 2002). As there are few options for treating cognitive deficits—inevitable in age—we think investigating performance-enhancing techniques, especially for an older age group, is of great practical relevance.

## 2 Material and methods

For comparability, the experimental procedures and behavioral data analysis followed the approach of Löffler et al. (2018) except for the extension of EEG and a 5-Hz-stimulation control group. The experimental protocol was approved by Medizinische Ethikkommission of the University of Oldenburg. Written informed consent was acquired from all participants prior to the experiment in line with the Declaration of Helsinki.

### 2.1 Participants

Forty-nine independent-living people aged 65 or older (mean age 72.4 years, *SD* 5.5, range 65–89) participated in the study. Participants received monetary compensation and were recruited from previous studies (not related to stimulation) and via a newspaper advertisement. Due to recording problems, one subject had to be excluded entirely from the analysis. Furthermore, reaction time recording failed in three subjects and the EEG recording failed in one subject, and they were excluded from the respective analysis. Therefore, only 45 (SHAM = 14, 5 Hz = 15, 40 Hz = 16) participants could be used for behavioral and 47 (SHAM = 15, 5 Hz = 14, 40 Hz = 18) for EEG analysis. Table 1 summarizes the group sizes and the characteristics of the remaining 48 participants. None of them reported the presence or history of neurological or psychiatric disorders. Twenty-eight subjects needed regular cardiovascular medication. Three subjects had vision impairment in one eye. Forty-five subjects were right-handed and three both-handed according to the Edinburgh handedness scale (Oldfield, 1971). One subject had experience with brain stimulation 4 years ago. One subject was remeasured after 18 months because sleep deprivation led to exorbitant reaction times (>2 s). Only the remeasurement was entered into the analysis.

**Table 1:** Group-wise sample size (total *n* = 48), mean age, and the number of female participants in total and according to the data analysis method.

**Group**	**SHAM**	**5 Hz**	**40 Hz**
**TOTAL**			
*n*	15	15	18
Age	71.7, *SD* 5.5	70.3, *SD* 3.6	74.3, *SD* 6.3
Female	8 (53%)	6 (40%)	9 (50%)
**Behavioral data analysis**			
*n*	14	15	16
Age	71.7, *SD* 5.7	70.3, *SD* 3.6	73.2, *SD* 5.3
Female	7 (50 %)	6 (40%)	8 (50%)
**EEG analysis**			
*n*	15	14	18
Age	71.7, *SD* 5.5	70.6, *SD* 3.4	74.3, *SD* 6.3
Female	8 (53%)	5 (36 %)	9 (50%)

EEG, electroencephalogram.

Participants were randomly assigned to one of the three experimental groups receiving 40-Hz, 5-Hz, or SHAM stimulation in a single-blind design. The groups were counterbalanced for participants' sex, age, and button press conditions. All participants believed they were receiving tACS stimulation and were debriefed after the experiment.

To select an appropriate sample size, we performed an *a priori* power analysis based on the findings of our study (Löffler et al., 2018), which suggested a sufficient power (1 – β = 0.85) at a total sample size of 42 (14 participants per group).

### 2.2 Procedure

**Figure 1:**
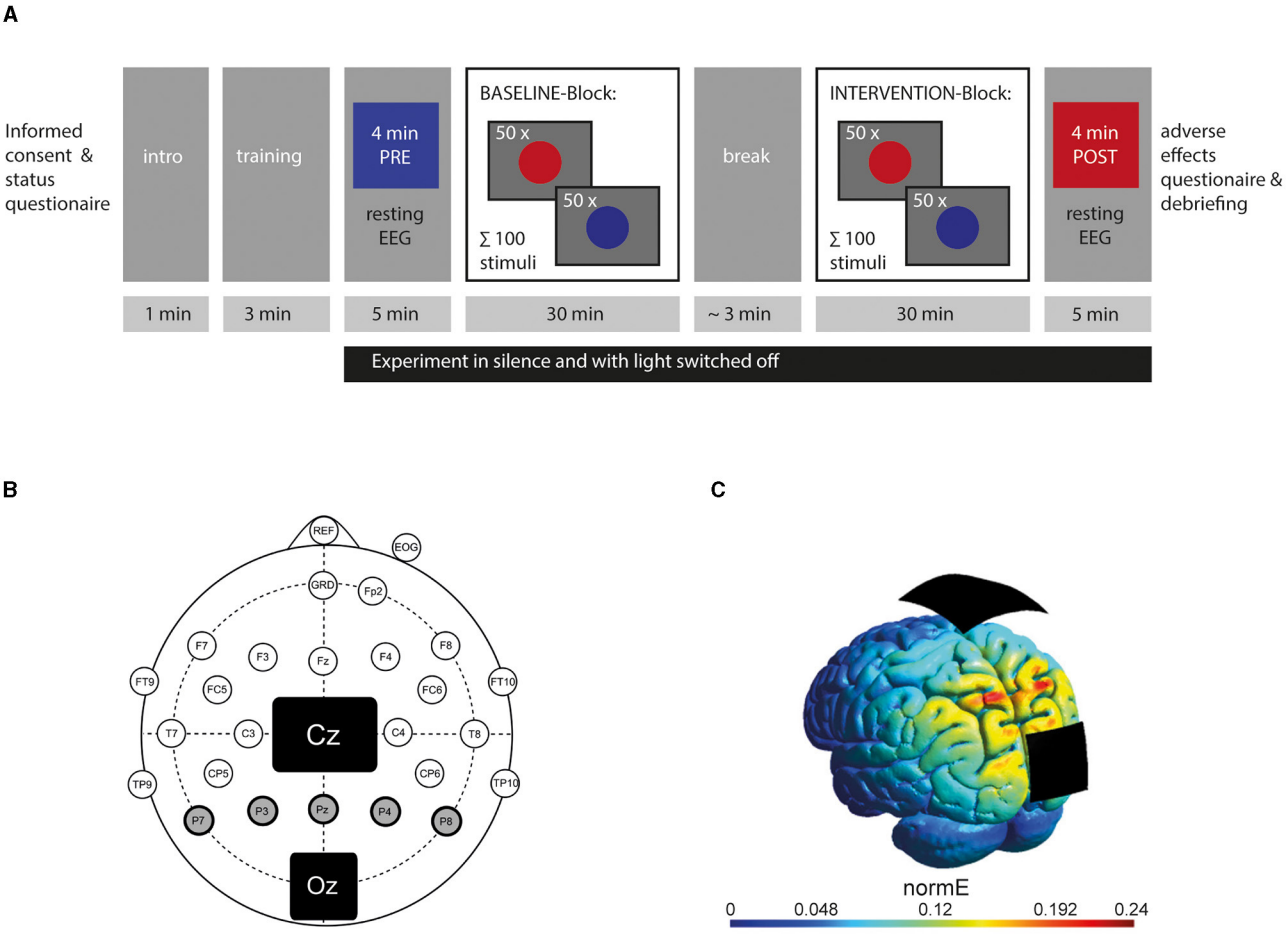
Design of the experiment: **(A)** Course of the experiment showing the timing of resting EEG-recording and behavioral records. **(B)** Electrode setup with a 5-cm × 7-cm stimulation electrode placed on the vertex (Cz) and a smaller 4.5-cm × 4.5-cm one on the visual cortex (Oz) according to the 10-10 system. Circles represent active electrodes used for EEG recording according to the modified 10-10 system. Posterior alpha power was calculated from five EEG electrodes (P7, P3, Pz, P4, P8) highlighted in gray with a bold line. **(C)** Current simulation using SIMNIBS showing the stimulation's electric field strength addressing the visual cortex (reproduced with permission of the authors from Stecher and Herrmann, 2018). The color bar represents the normal vector of the electric field in V/m.

The experiment took place in a laboratory room, where participants were seated on an office chair at a distance of approximately 90 cm in front of a 24-in. computer screen (60 Hz, 1,920 × 1,800 px resolution) and connected to the EEG and stimulation device. Before the experiment, participants were given an introduction and a small training session to avoid confusion. During the experiment, participants and examiner were separated via a gray screen, communication stopped, and the light was switched off. Participants were not informed about the time of tACS onset. After the experiment, participants were asked if they believed to be stimulated or not and were informed about the actual stimulation settings applied to them. Furthermore, they were asked to fill out a standardized questionnaire on adverse effects according to Brunoni et al. (2011). Questions contained the common 10 side effects as *headache, neck pain, itching, tiredness*, and others and the link to being stimulated or not. Items were on a scale of 1 (*none*) to 4 (*severe* or *definitely*, respectively). See Figure 1A for the course of the experiment.

### 2.3 Study design

The experiment consisted of two 30-min-long blocks with a baseline block (referred to as the BASELINE block) and a stimulation block (referred to as the INTERVENTION block) in which participants were stimulated according to their group (SHAM, 5-Hz, or 40-Hz tACS). To ensure the presence of a vigilance decrement, the block order was not randomized, and no participant received stimulation during the BASELINE block. Therefore, all participants irrespective of group affiliation should show similar behavioral outcomes during the BASELINE block, while differentiation into the stimulation groups should only be relevant in the INTERVENTION block. Before the BASELINE and after the INTERVENTION block, a 5-min-long resting EEG was recorded, and participants were asked to sit still, relax, and keep their eyes open. This time span has been used in similar studies by other researchers investigating EEG in older adults (Babiloni et al., 2015; Scally et al., 2018; Rumpf et al., 2019; Varastegan et al., 2023). During an approximately 2–3-min-long self-paced break (mean 148 s, *SD* 66 s) between the blocks, the participants were allowed to drink and rest.

### 2.4 Task

Participants were asked to put their left and right index fingers on the respective buttons of a custom-made, software-debounced button box placed on the table before them and asked to fixate a white cross (10 × 10 px) displayed on a gray (RGB = 95 95 95) background. Every 6–56 s (median: 17 s), either a red (RGB = 240 55 55), or a blue (RGB = 20 100 255) stimulus in the shape of a circle (400-px diameter) appeared for 500 ms at the center of the screen. Half of the participants of each group were instructed to press the left button when the red stimulus and the right button when the blue stimulus appeared (the other half vice versa) as fast and correctly as possible. A specific set of 100 stimuli was used (50 red, 50 blue) in a pseudo-randomized order for each block. Stimulus presentation was handled with the Psychophysics Toolbox extension (version 3.0.12) and button presses recorded in MATLAB (Release 2012a, The MathWorks Inc., Natick, MA, United States).

### 2.5 EEGs

EEGs were acquired with a rate of 10 kHz from 23 active electrodes using an actiCHamp amplifier (Brain Products GmbH, Gilching, Germany) and recorded via Pycorder software (Brain Products GmbH, Gilching, Germany). The 10-10 system was used to place the electrodes (see Figure 1B for an overview of recording channels), omitting the sites of the stimulation electrodes. The ground electrode was positioned at FPz. An electrode attached to the nose was chosen as a reference since it is widely used in neural research addressing visual cognition as the electrodes of interest at the visual cortex are far away from the nose (e.g., Helfrich et al., 2014a,b; Kasten et al., 2016). A vertical electrooculogram was recorded underneath the right eye to monitor eye movements. All impedances were kept below 20 kOhm.

### 2.6 tACS

Two rubber electrodes were positioned with their center at Cz (7 × 5 cm^2^) and Oz (4.5 × 4.5 cm^2^) according to the 10-10 EEG system and fixated with adhesive electrode paste (ten20^®^conductive, Weaver and Company, Aurora, CO, USA). A battery-driven stimulator was used (neuroConn DC Stimulator with Remote-In function, Neuroconn GmbH, Ilmenau, Germany), and impedance was kept below 10 kOhm. For the STIMULATION block, the tACS device was remotely accessed via a MATLAB-controlled DAQ module (Ni USB 6229, National Instruments, Austin, Texas, USA). Stimulation intensity was set to 1 mA. For the 5-Hz and 40-Hz group, the current was linearly faded in and out for 30 s at the beginning and end of the INTERVENTION block. Current in the SHAM group was faded in for 30 s, kept constant at 1 mA (at either 40 or 5 Hz) for 30 s, and faded out for 30 s at the beginning and end of the INTERVENTION block. Electrode sizes, positions, and stimulation intensity were in line with the effective montage used in our study (Löffler et al., 2018) and by other studies, in which stimulation affected alpha bands in the visual cortex (Kasten et al., 2016; Stecher et al., 2017). The intensity of 1 mA has been proven to induce neurophysiological and behavioral effects without causing unpleasant feelings by the same studies and is a widely used stimulation intensity (e.g., Klink et al., 2020b). Figure 1C shows a computer simulation of the tACS setup and its induced electric field addressing the visual cortex. tACS at 40 Hz has been shown to successfully downregulate alpha power at the visual cortex (Helfrich et al., 2014a, 2016).

### 2.7 Data processing

Data processing and analysis were performed using MATLAB (Klink et al., 2020a) and the Fieldtrip toolbox (Oostenveld et al., 2011).

Initial preprocessing of the behavioral data was done in MATLAB. Missed stimuli, wrong button presses, and reaction times below 200 or above 2,000 ms were excluded from reaction time analysis and considered “errors.”

The EEG data was down-sampled to 500 Hz and high-pass filtered at 1 Hz. We used a 48-Hz low-pass filter to remove line noise and high frequency (e.g., muscle artifacts). Two 4-min chunks of resting EEG (PRE: before and POST: after the experiment) starting 30 s after the beginning of the respective resting EEG measurement were cut into trials of 1 s to easily help identify and reject artifactual segments. These trials were then used in an independent component analysis approach. The identification of ocular components was based on topography (frontal and fronto-lateral bipolar) and time course (strong sigmoid shapes and boxcar shapes). Components containing vertical or horizontal eye movements were manually removed. Furthermore, we removed trials containing voltage differences >200 μV in the Pz electrode signal. On average, 230.23 out of 240 s were left after threshold-based rejection (STD: 16.18, min: 154).

As occipital electrode positions were covered by the stimulation electrodes, posterior alpha power was estimated using all parietal electrodes from both hemispheres (Pz, P3, P4, P7, P8) except for one participant, for whom P4 and P7 were excluded due to excessive noise. A fast Fourier transform using a Hanning window with 5-s zero padding was computed on the data. The results were 1–48-Hz-long bands of 236 data points with a resolution of 0.2 Hz. We calculated the mean value of all P-electrodes. We used the MATLAB function *findpeaks* to find the maximum power value between 6.8 and 13 Hz and its respective peak frequency, the individual alpha frequency (IAF). We defined alpha power as the mean power value +/– 1 Hz around peak frequency.

### 2.8 Statistical analysis

The software R 4.0.3 (R Foundation for Statistical Computing, Vienna, Austria) was used for statistical analysis.

For statistical analysis of the behavioral data, a linear mixed model (LMM) with the add-on-package “nlme” (Pinheiro et al., 2022) for reaction time and a generalized additive mixed model (GAMM) of the add-on package “mgcv” (Wood, 2017) for error analysis were used. In line with the statistical analysis used in our previous study (Löffler et al., 2018), data were separated into six groups, with the indexes _base_ and _inter_ indicating the block (BASELINE or INTERVENTION): SHAM_base_, 5 Hz_base_, 40 Hz_base_, SHAM_inter_, 5 Hzinter, 40 Hz_inter_. SHAM_base_ and SHAM_inter_, 5 Hz_base_ and 5 Hz_inter_, and 40 Hz_base_ and 40 Hz_inter_ consisted of the same participants, respectively. We expected the three base groups (SHAM_base_, 5 Hz_base_, and 40 Hz_base_) to show similar outcomes as no participant was stimulated during BASELINE block. Fixed factors were *group* (indicating the six groups as mentioned earlier), *time*, and the interaction term of both *group* ^*^ *time*. The SHAM_base_ group served as the base group to which the other five were compared. The continuous variable *time* represented the pseudo-randomized stimuli onset time point (100 per block) starting from zero in each block. As random effects, individuals and their variation of reaction times or error probability over time were used. We increased complexity stepwise (intercept to broken-stick models with 8 degrees of freedom). We checked for models' improvement using likelihood ratio tests of the R package “performance” (Lüdecke et al., 2021) for the LMM and Akaike information criterion (AIC) for the GAMM. A two-sided significance level of α = 0.05 was used.

For the LMM, a simple linear model with a continuous autoregressive covariance structure best described the observed effects. We also checked the influence of other factors, such as *gender* as fixed or *age* and *IAF* as random factors, but these did not improve the model's fit. The literature shows that tACS is often more beneficial for a specific subgroup of participants, for example, the influence of baseline performance (Santarnecchi et al., 2016; Thompson et al., 2021) or in participants showing a low level of arousal (Martínez-Pérez et al., 2022). Therefore, we defined a subset of subgroups (classifying participants into two groups by using the median) that we integrated as fixed effects. While *slow performance, IAF level*, and *alpha power difference* showed no effect, we found a significant contribution to the model for *ageclass* (younger than the median age of all participants of 72 years or equal and older) and *medication* (yes/no) over *time* and *group*. While *ageclass* seemed only to show variation and was not interpretable, *medication* attributed to our model. But, as *medication* was not part of our hypothesis and not balanced over groups, we feared overfitting. A more extensive data set is needed to make reliable statements about this factor. In tDCS studies, *medication* has significantly influenced the stimulation effect (McLaren et al., 2018).

Concerning errors, we were not able to fit a satisfying mixed model. Random effects improved the fit of the GAMM, but introducing covariates did not. We could not find a suitable model (conditional *R*^2^ < 0.3) and any linear effect of time.

The EEG data's mean power values PRE and POST were not normally distributed according to Shapiro–Wilk test. QQ and density plots of the data showed the uneven distribution of the data, with a concentration of small power values at approximately 1 μV^2^, while some participants showed high power values of 10 μV^2^ and more. Due to this huge inter-individual variance but intra-individual-specific voltage level, we decided on a statistical analysis with a generalized linear mixed model (GLMM) using the gamma-distribution family to describe the data. We used the R package “lme4” (Bates et al., 2015). We applied *treatment* (SHAM, 5 Hz, and 40 Hz) and *timepoint* (PRE and POST) as fixed factors. Increasing the models' complexity stepwise, introducing different link functions, and comparing AIC values showed a GLMM with individual random effects and a log-link function, but excluding the factor *treatment* served best to describe the data.

As not only the absolute power values of each participant but also the difference between POST and PRE alpha power were relevant for this study, we calculated a Wilcoxon rank-sum test to show the effect size of the differences between POST and PRE alpha power.

We calculated linear regression models testing different correlations during the BASELINE block to support theories about our cohort's behavior and neurophysiological nature. We tested *age, mean reaction time, mean-variance of reaction times, amount of errors, alpha power differences, IAF*, and *alpha PRE power* (block-wise and in total, where appropriate) against each other. Shapiro–Wilk tests were used for assessing normal distributions of the data sets. Kendall *r* was used as the correlation coefficient for non-normally distributed data sets; for normally distributed ones, Pearson's *r* was used.

## 3 Results

### 3.1 Debriefing

One participant (SHAM group) did not answer the questionnaire about side effects and stimulation. The side effects mentioned most frequently by the remaining 47 participants (intensities rated higher than 1) were *tiredness* (47%) and *trouble concentrating* (66%), but only 15% *(tiredness*) or 13% (*concentration*) of the participants attributed it to being stimulated. Other side effects mentioned by more than three participants were *tingling* and *itching* both mentioned by 7 out of 47 of the participants (15%). Of the subjects, 35% (33% of the SHAM, 21% of the 5-Hz, and 44% of the 40-Hz group) thought they were stimulated (*n* = 46, one participant of the 5-Hz group did not answer this question).

### 3.2 Behavioral analysis

#### 3.2.1 Reaction times analysis

**Figure 2:**
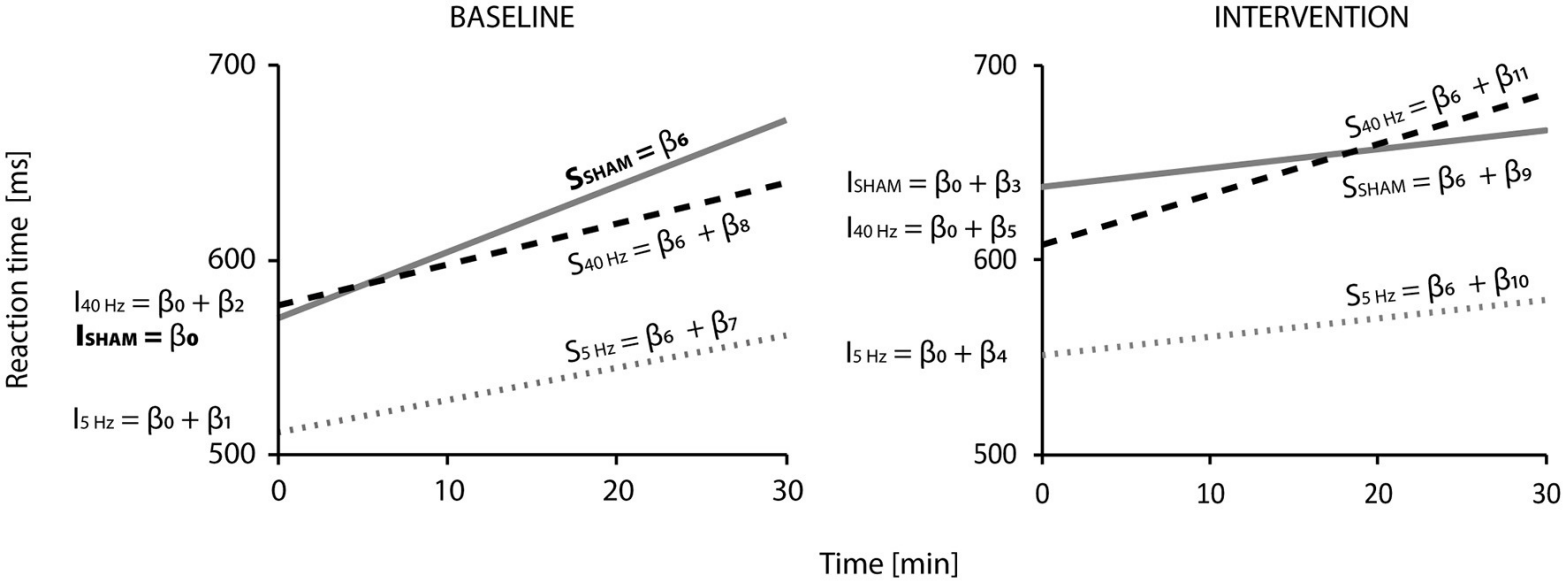
Linear mixed model results for reaction times (*n* = 45, observations = 8,413): Visualization of the regression functions representing reaction time (in ms) against time (in min) for the SHAM (gray solid line), 5-Hz (gray pointed line), and 40-Hz (black dashed line) according to block (BASELINE or INTERVENTION). Regression lines represent the six groups (BASELINE: SHAM_base_, 5Hz_base_, 40Hz_base_; INTERVENTION: SHAM_inter_, 5-Hz_inter_, 40-Hz_inter_). The two SHAM, 5-Hz, and 40-Hz-groups consisted of the same participants. β-coefficients (from Table 2) indicate how intercepts—indicated by I (β_0_-β_5_) and slopes—indicated by s – (β_6_-β_11_) were calculated. β_0_ is the intercept of the SHAM_base_ group, and β_6_ is its increase over time—their significance is compared to zero and shows a highly significant difference (*p* < 0.001). All other intercepts and slopes are compared to SHAM_base_: Significant differences according to the model can be found for the intercept of the SHAM_inter_ group (I_SHAM_ = β_0_ + β_3_; *p* < 0.001) and the slopes of the SHAM_inter_ (S_SHAM_ = β_6_ + β_9_) and 5-Hz_inter_ (S_5Hz_ = β_6_ + β_10_; *p* < 0.05) groups. Model parameters are *R*^2^ marginal = 0.056, *R*^2^ conditional = 0.470, and Akaike information criterion = −9,055.448.

The LMM predicting reaction times shows a slight increase over time, higher in the BASELINE block. In the INTERVENTION block, initial reaction times (intercepts) are higher than in BASELINE, and the slopes for the SHAM_inter_ and 5-Hz_inter_ groups show a less steep increase, while the one for the 40-Hz group shows a steady one (see Figure 2).

Reaction times are predicted according to the following Equation 1 from the covariates *group* (SHAM_base_, 5 Hz_base_, 40 Hz_base_, SHAM_inter_, 5 Hz_inter_, 40 Hz_inter_), *time*, and *group* ^*^ *time* (interaction term):


(1) $$\begin{array}{l} \text{rt = }{\text{β}}_{0}{\;}^{*}\text{SHA}{\text{M}}_{\text{base}}\text{+ }{\text{β}}_{\text{1}}{\;}^{*}\text{5}Hz_{base}\text{+ }{\text{β}}_{\text{2}}{\;}^{*}\text{40 }Hz_{base}\text{+ }{\text{β}}_{\text{3}}{\;}^{*}SHAM_{inter}\\ \text{ + }{\text{β}}_{\text{4}}{\;}^{*}\text{5}Hz_{inter}\text{ + }{\text{β}}_{3}{\;}^{*}\text{40}Hz_{inter}\text{ + }{\text{β}}_{6}{\;}^{*}\text{SHA}{\text{M}}_{\text{base}}*\text{time}\\ \text{ + }{\text{β}}_{\text{7}}{\;}^{*}\text{5}Hz_{base}\text{*}time\text{ + }{\text{β}}_{\text{8}}{\;}^{*}\text{40}Hz_{base}\text{*}time\text{ + }{\text{β}}_{\text{9}}{\;}^{*}SHAM_{inter}{\;}^{*}time\\ \text{ + }{\text{β}}_{\text{10}}{\;}^{*}\text{5}Hz_{inter}{\;}^{*}time\text{ + }{\text{β}}_{\text{11}}{\;}^{*}\text{40}Hz_{inter}{\;}^{*}time. \end{array}$$

β coefficients represent fixed effects and are listed in Table 2. β_0_ describes the intercept of the SHAM_base_ group and β_6_ its slope (i.e., increase of reaction time over time). *p*-values for the SHAM_base_ group indicate the difference between zero (bold font). The coefficients β_1_-β_5_ describe the group-specific difference of intercepts compared to SHAM_base_ group (β_0_), and β_7_-β_11_ are their slopes. For other groups, respective coefficients need to be added to the ones of the SHAM_base_ group. For example, (β_0_ + β_1_) add up to the intercept of the 5-Hz_base_ group, (β_0_ + β_2_) to the intercept of the 40-Hz_base_, and so on. For coefficients, β_1_-β_5_ and β_7_-β_11_, *p*-values indicate the significance of the difference compared to the SHAM_base_ group (β_0_ und β_6_). Figure 2 visualizes the resulting regression functions. The final model, including fixed and random effects, explains approximately 47% of the observed variance (*R*^2^ conditional = 0.470).

**Table 2:** Linear mixed model results for reaction times (*n* = 45, observations = 8,413): β presents the regression coefficients; *SE* β, the standard error of β.

	**β**	***SE* β**	***t*-value**	***p*-value**	
**Intercepts**					
**β_0_ SHAM_base_**	**569.9**	**28.9**	**19.70**	**0.000**	**<0.001** ^°***^
β_1_ 5 Hz_base_	−58.2	40.3	−1.45	0.149	>0.05
β_2_ 40 Hz_base_	7.3	39.6	0.18	0.855	>0.05
β_3_ SHAM_inter_	67.6	11.9	5.67	0.000	<0.001^***^
β_4_ 5 Hz_inter_	−18.7	40.2	−0.47	0.642	>0.05
β_5_ 40 Hz_inter_	37.6	39.6	0.95	0.342	>0.05
**Slopes**			
**β_6_ time ×SHAM_base_**	**3.403**	**0.698**	**4.88**	**0.000**	**<0.001** ^°***^
β_7_ time × 5 Hz_base_	−1.746	0.974	−1.79	0.073	>0.05
β_8_ time × 40 Hz_base_	−1.320	0.955	−1.38	0.166	>0.05
β_9_ time × SHAM_inter_	−2.424	0.700	−3.46	0.001	<0.01^**^
β_10_ time × 5 Hz_inter_	−2.448	0.965	−2.54	0.011	<0.05^*^
β_11_ time × 40 Hz_inter_	−0.810	0.959	−0.85	0.395	>0.05

β_0_ represents the initial reaction time, and β_6_ the increase in reaction time over time of the SHAM_base_ group (in bold). Their *p*-values, indexed with a °, show a significant difference compared to zero. All other coefficients are tested against β_0_ and β_6_. Intercepts are given in ms, slopes in ms/min. ^***^p-value < 0.001, ^**^p-value < 0.01 and ^*^p-value < 0.05.

According to the LMM, initial reaction times were approximately 570 ms (β_0_) in the SHAM_base_, 512 ms in the 5-Hz_base_ (β_0_ + β_1_), and 577 ms (β_0_ + β_2_) in the 40-Hz_base_ groups at the beginning of the experiment. In the BASELINE block, reaction times of the SHAM_base_ group increased significantly with 3.4 ms/min (β_6_, *p* < 0.001) compared to zero. The expected reaction time at the end of the BASELINE block and after 30 min added up to 672 ms (which results in a difference of Δ102 ms—the Δ symbol marks the value as a difference) for the SHAM, 562 ms (Δ50 ms) for the 5-Hz, and 640 ms (Δ63 ms) for the 40-Hz groups. This equalizes to a rise of 18, 10, and 11%, respectively. There was no statistical difference among the BASE groups (although the slope of the 5-Hz_base_ group nearly touched significance with a *p*-value of 0.073).

All participants started the INTERVENTION block higher than their group-specific initial reaction time but lower than they finished the BASELINE. Participants of the SHAM group started Δ 68 ms; of the 5-Hz group, Δ40 ms; and of the 40-Hz group, Δ31 ms slower than they began in BASELINE. The difference between the expected reaction time at the end of BASELINE compared to the start in INTERVENTION was Δ-34 ms for the SHAM group, Δ-10 ms for the 5-Hz group, and Δ-32 ms for the 40-Hz group. In comparison to SHAM_base_, the intercept of the SHAM_inter_ group showed a significant difference (*p* < 0.001). During block 2 (INTERVENTION), the increase of reaction times slowed down for the SHAM_inter_ group (by Δ2.42 ms/min) and the 5-Hz_inter_ group (by Δ0.70 ms/min) to approximately 1 ms/min. The 40-Hz_inter_ group showed a slight increase of Δ 0.51 ms/min to 2.5 ms/min. Compared to SHAM_base_, the change was significant for the SHAM_inter_ and 5-Hz_inter_ groups (*p* < 0.05) but not for the 40-Hz_inter_ group. The rise during the INTERVENTION block equalizes to 5% for the SHAM and 5-Hz groups (~Δ30 ms) and 13% (Δ78 ms) for the 40-Hz group. At the end of the INTERVENTION block, the expected reaction times are 667 ms for the SHAM group, 580 ms for the 5-Hz group, and 685 ms for the 40-Hz group.

We also calculated models with 5 Hz and 40 Hz as the base group to test the difference between base and inter-conditions. See the Supplementary material for more details. We confirmed that 5-Hz_base_ and 5-Hz_inter_ intercepts and the 40-Hz_base_ and 40-Hz_inter_ intercepts differ significantly from each other: All groups started the INTERVENTION block significantly slower compared to their initial reaction times (5-Hz_base_ intercept vs. 5 Hz_inter_: *p* < 0.001, 40-Hz_base_ intercept vs. 40 Hz_inter_: *p* < 0.01). No significant difference in slopes could be detected for both models.

#### 3.2.2 Error analysis

It was impossible to fit a GAMM explaining a substantial amount of variance (*R*^2^ = 0.000898) to the error data, so no change in the error occurrence probability could be detected over time. Mean performance was 93% (*SD* 5%, range 78–99%).

### 3.3 EEG

**Figure 3:**
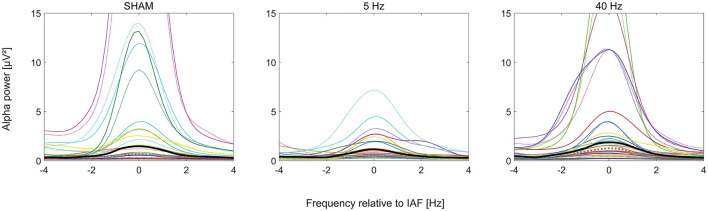
Plots of the alpha power (*n* = 47) spectrum in μV^2^ normalized to individual alpha frequency in Hertz according to group (SHAM, 5 Hz, and 40 Hz). The dotted curves show PRE, solid POST alpha power. Bold curves show the median alpha power of all participants per group (PRE: gray, POST: black). Individual participants' curves are represented by individual colors.

Mean IAF was 8.9 Hz (*SD* 1 Hz). Comparing PRE and POST alpha power, 78% of the participants showed an increase in alpha power after the experiment (Figure 3)—irrespective of group (SHAM = 80%, 5 Hz = 71%, 40 Hz = 82%). The best-fitting GLMM (gamma distribution with log link) predicts alpha power with participant ID as a random effect (intercept) and *timepoint* as the only covariate according to Equation 2:


(2) $$\text{alpha power = exp}({\text{β}}_{\text{0}}+\;{\text{β}}_{\text{1}}{\;}^{*}\;\text{timepoint}).$$

Coefficients are log-scaled (β_0_ = −0.00113, β_1_ = 0.313). Back transferred, alpha power PRE is predicted to be 1 μV^2^ and POST to be 1.37 μV^2^. According to the model, the increase in POST was significantly different from zero (*p* < 0.001). The model's AIC is 166.2, with *R*^2^ marginal = 0.021 for the fixed and *R*^2^ conditional = 0.838 for the random and fixed effects.

**Figure 4:**
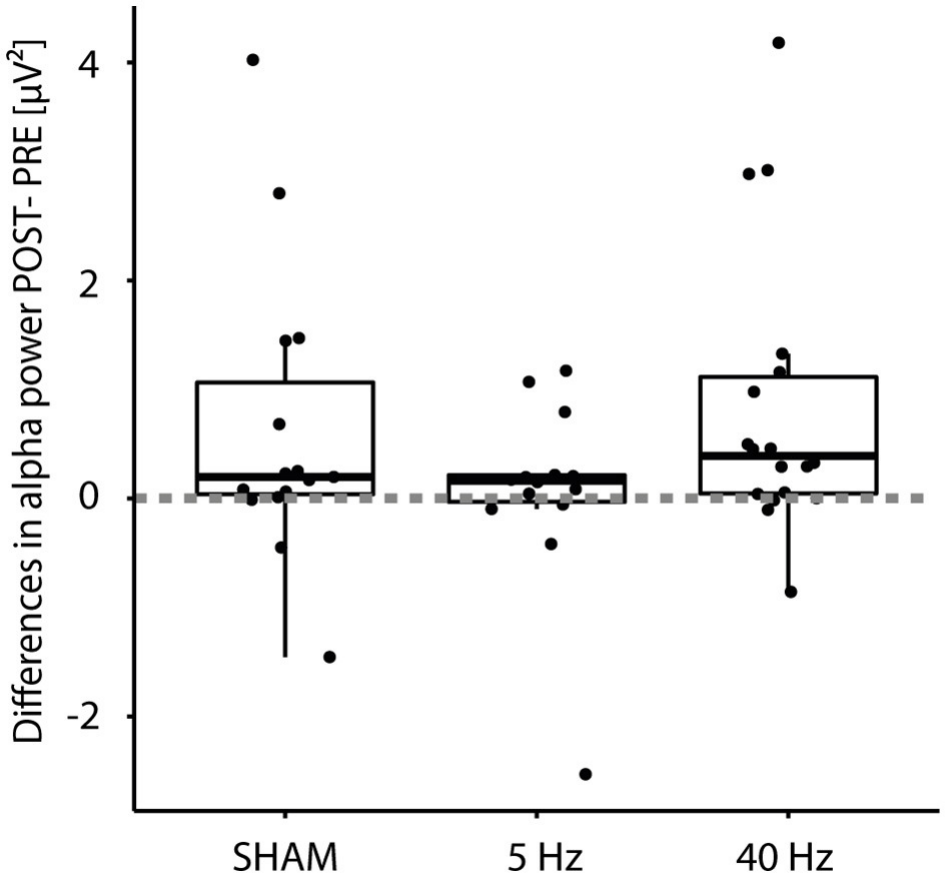
Boxplot of differences (POST – PRE) in alpha power (*n* = 47) according to *Treatment* group (SHAM, 5 Hz, and 40 Hz). Filled dots indicate individual measurements; the dashed line indicates the zero crossing.

We also looked at the absolute differences in alpha power (POST–PRE): A one-sided Wilcoxon rank-sum test showed a significant difference (*p* < 0.001) with an effect size of 0.55 (strong; see Figure 4). A Kruskal–Wallis Test testing differences in alpha power between the three treatment groups revealed no significance (*p* = 0.3).

### 3.4 Regression analysis

A modest significant linear correlation could be obtained for *IAF* vs. *errors* (Kendall *r* = −0.22, *p* = 0.046) and *IAF* vs. *mean variance of reaction times* (Kendall *r* = −0.23, *p* = 0.034) and a strong one for *mean reaction time* vs. *mean variance of reaction times* (Kendall *r* = 0.74, *p* < 0.001). A positive linear tendency (*r* > 0.2) was detected for *mean reaction time* vs. *age*, the *mean variance of reaction time* vs. *age*, and the *mean variance of reaction time* vs. *errors* and a negative tendency (*r* > −0.2) for *alpha power* vs. *age* and *IAF* vs. *mean reaction times*, but these correlations were not significant.

## 4 Discussion

Aging is accompanied by growing cognitive deficits (Grady, 2012). In the eye of demographic change, enhancing the cognitive performance of older adults becomes essential. One aspect of cognitive performance is vigilance, which plays a critical role in professional and private settings where tasks can be safety-relevant but tiring (Warm et al., 2008). We, therefore, repeated our study (Löffler et al., 2018) in which we applied gamma tACS to counteract vigilance decrement but with older adults. We hypothesized that participants treated with 40-Hz tACS would exhibit less increase in reaction time increment and have a lower alpha power level after the experiment compared to SHAM and 5-Hz control groups. Results of reaction time slowing and higher POST alpha power indicate that we succeeded in inducing vigilance decrement (Buck, 1966; Molina et al., 2013) but failed to show an effect of our intervention—and if then only a trend in reaction time increment but in the opposite direction as expected. In the following, we discuss our results and give possible explanations.

### 4.1 Anatomical and functional differences of the aging brain

There is some debate about the nature of brain aging, it is generally agreed that aging leads to a loss of brain volume and further physiological changes especially considering alpha and slow rhythms (Hedden and Gabrieli, 2004; Ishii et al., 2017). These changes could be critical for the success of tACS, which strongly depends—among others—on internal frequencies and the electrical field strength of the addressed tissue. In line with this, one recent tACS study addressing older adults used magnetic resonance imaging (MRI) scans to model individual electric fields and correlate them to the success of tACS together with EEG measuring the closeness of stimulation frequency (6 Hz) to internal brain waves (theta peak; Zanto et al., 2021). The researchers found that performance change correlates to the modeled electric fields and frequency mismatch. This explained 54–65% of the variance in tACS-related performance improvements. Important: On a group level, it was not possible for them to find any significant stimulation effect.

We did not measure the electrical field strength in our participants and can only speculate. Research has shown that brain volume seems to greatly decrease within the anterior regions while neural loss is rarely observed in occipital regions (Raz et al., 2005). So we still addressed—on the tissue level—a probable intact region. However, in aging, activation seems to shift from posterior to anterior areas (Dennis and Cabeza, 2011). Regarding alpha oscillations, many studies observed a significant alpha increase in frontal regions (Ishii et al., 2017). This activation shift has been associated with a compensatory mechanism (Mattay et al., 2002) and hyperactivation (Berchicci et al., 2012). Thus, it is conceivable that the visual cortex is not the relevant stimulation site for aged brains, as has been shown for stimulus processing and formulated in the posterior–anterior shift in aging hypothesis (Dennis and Cabeza, 2011).

Considering posterior alpha oscillations, it is generally agreed that older adults show a lower IAF, a marked reduction in amplitude, and declined reactivity (Babiloni et al., 2006; Ishii et al., 2017; Knyazeva et al., 2018). Although the general working principle of tACS is not completely understood, it is generally believed that tACS works as an external oscillator entraining internal brain oscillations (Vosskuhl et al., 2018). Therefore, stimulation success is dependent on selecting the correct stimulation site, electrode size, duration, frequency, and intensity according to the intrinsic properties of the addressed brain (Herrmann et al., 2013).

The mean IAF of the older subjects in our study was 8.9 Hz (*SD* 1 Hz), which is slightly less but still comparable to values from the literature (Klimesch, 1999; Barry and de Blasio, 2017). The average IAF obtained for a younger target group with the same equipment and measurement in our laboratory group at electrode Pz was 10 Hz (*SD* 0.3 Hz; Stecher et al., 2021). The variance was smaller, and the IAF was closer to the optimal 10 Hz, a subharmonic of the 40 Hz stimulation frequency and probably needed to induce CFC. We assume that our 40-Hz stimulation frequency was too far a mismatch from the lower IAF in older adults to induce any behavioral or physiological effect.

In physiological aging, alpha sources in posterior regions seem to have significantly less magnitude compared to young ones (Babiloni et al., 2006; Ishii et al., 2017). As the power level of the addressed intrinsic frequency is important for successful stimulation, lower power could have influenced the stimulation outcome. It has been shown that alpha tACS can only enhance IAF amplitude if the initial power level is low (Neuling et al., 2013). We observed alpha power of more than 15 μV^2^ in seven participants; others showed only marginal power values of approximately 0.2 μV^2^. This is in line with the literature because alpha power can show a high inter-individual variability due to anatomic and genetic differences among people (Bazanova and Vernon, 2014; Haegens et al., 2014). Comparable studies measured 0.8–1 μV^2^ for participants with an average age of 69 years (Vaden et al., 2012; Barry and de Blasio, 2017).

We integrated IAF and power level in our LMM and performed further linear correlation analyses but could not detect any effect.

### 4.2 Motivation and difference in mindsets

We did not document the motivation or arousal level in our study, but higher motivation—as compared to younger participants—is a well-documented phenomenon in studies with older adults (Tomporowski and Tinsley, 1996). We can only speculate, but it may have been possible that at least part of the tested group of older adults experienced mental depletion instead of boredom. As mental depletion is associated with frontal brain regions in older adults (Arnau et al., 2017), the visual cortex might again not have been the correct stimulation area for inducing a performance change. Other tACS studies also show that mindsets are critical for stimulation success (Mierau et al., 2017).

### 4.3 Heterogeneity

Due to differences in scalp and skull thickness, hairs, and IAF, it is hard to repeat the effects of stimulation in a younger group, which, in general, consists of 20–30-year-old students (Kasten et al., 2019). With growing age, heterogeneity increases due to different life experiences and styles, nutrition, education, cognitive fitness, agility, medication and illnesses, and probably even more aspects (Light et al., 1996)—digital media use to name one more (Taipale et al., 2021). Compared to the repeated study (Löffler et al., 2018), the age range of the target group was higher (65–89 years compared to 20–30 years), as well as the educational background.

Next, to the already mentioned IAF and alpha power, this is reflected by reaction times and power differences: Fast-performing participants showed mean reaction times of 450 ms and small variance; others needed approximately 1 s to make a choice: Reaction times become more variable with age (Hultsch et al., 2002; Gorus et al., 2008). While alpha power decreased in roughly one-third of the participants (three in the SHAM group, four in the 5-Hz group, and three in the 40-Hz group; see Figure 4), seven participants showed a substantial increase of 200% and more (two in the SHAM group, three in the 5-Hz group, and two in the 40-Hz group). These inter-individual differences were irrespective of group. Linear regression analysis showed a significant interaction between mean reaction time and reaction time variance, as known from the literature (Welford, 1971). Inter-individual differences seem consistent: The groups' mean reaction times differed, with the 5-Hz group showing the fastest. We nearly observed a significant difference between the 5-Hz group and the other groups in the BASELINE block for intercept and slope, which we did not expect, because participants were selected to be out of the same basic population. This performance gap continues in the INTERVENTION block, with a lower mean reaction time but not in the slope compared to the SHAM group. Group differences are also reflected in alpha power, with candidates of the 5-Hz group showed no power values of 10 μV^2^ and more.

Still, our data show that we were able to induce vigilance decrement as expected by a constant reaction time slowing with time on task and a higher alpha power level post the experiment: Reaction times of approximately 545 ms are in close range of similar experiments with older adults (Welford, 1988) but slower than in the previous study (Löffler et al., 2018), with an average initial reaction time of approximately 485 ms. The reason for slower reaction times can be not only physiological (Welford, 1988) but also because of a different mindset: Older adults tend to be more careful and do not like to make mistakes (Botwinick, 1966). As expected, participants started the INTERVENTION block with a significantly higher initial reaction time compared to their initial BASELINE time. The break between blocks led to a slight recovery—a well-known effect (Ross et al., 2014) that has been also observed with young participants (Löffler et al., 2018). The less steep slope in the INTERVENTION block might be due to a ceiling (Neuling et al., 2013) or learning effect (Arnau et al., 2017; Getzmann et al., 2018).

No pattern of error-making could be detected nor any difference between the blocks: This is consistent with previous findings (Löffler et al., 2018), confirming that making mistakes is not critical to good performance (Sarter et al., 2001) and fits with the idea mentioned earlier that older adults are slower but also more careful.

Despite not being part of our hypothesis, we want to quickly discuss the factor *medication*. Of our 48 participants, 28 needed regular cardiovascular medication known to influence stimulation effects (McLaren et al., 2018). In the LMM not considering the factor *group*, people on *medication* suffered significantly less from reaction time increment. In the LMM with the factor *group*, people on *medicatio*n suffered significantly less from reaction time increment as well, but this effect was significantly strengthened only in the INTERVENTION block for the 40-Hz group. This indicates that 40-Hz tACS was especially beneficial for people on cardiovascular treatment (see the Supplementary material). Due to the small sample size, we can only speculate but still want to emphasize the influence of medication on tACS findings.

The model presented in our Results section shows a significant difference between groups. Although alternative versions of the model (see the Supplementary material) indicate that this significance is only due to the high interindividual variance, we still want to discuss this point as other tACS studies comparing young and old participants also found opposing effects of stimulation between age groups. In one study (Rufener et al., 2016), stimulating the auditory cortex with 40-Hz tACS to improve performance-diminished task accuracy in young adults, whereas older adults benefited from the stimulation. No behavioral differences were found in the 6-Hz condition between both age groups. Here, 40-Hz tACS was applied to the bilateral auditory cortex to counteract age-dependent changes of gamma oscillations relevant for processing temporal features of spoken language. They argue that in young adults, gamma oscillations are optimal and that introducing more energy via tACS perturbs the balance, leading to the observed inaccuracy. Another study reports a positive effect of IAF tACS on general motor skills and sequence-specific skill consolidation in an old target group, while the same stimulation parameters were detrimental for the young group (Fresnoza et al., 2020). The researchers argue that this opposing effect might be due to the age-related difference in the electrical field of older adults' brains which is in general not as conductive as a younger brain. As a consequence, only a comparably low stimulation intensity reaches the relevant brain tissue that—as known from studies stimulating with direct current—shows inhibitory effects while high intensities act excitatory (Moliadze et al., 2012; Batsikadze et al., 2013). We observed a similar behavior in our control groups (SHAM and 5 Hz), which supports the notion that stimulation with 5 Hz on the visual cortex shows no effect and works as a control frequency. The opposing behavior of the 40-Hz-stimulated group might be due to age-dependent changes in the brain, possibly due to inhibitory effects of the stimulation like Fresnoza and colleagues (2020) argue or because of changes or disturbances of internal gamma (or other) oscillations like in Rufener et al. (2016)—preventing CFC and the down-modulation of the alpha amplitude via 40-Hz tACS and distracting behavioral optimization processes otherwise observed in the control groups.

In general, most studies stimulating older adults with a visible effect of stimulation-applied intensities of 1.5 mA or higher (Rufener et al., 2016; Borghini et al., 2018; Reinhart and Nguyen, 2019; Fresnoza et al., 2020; Benussi et al., 2021; Kim et al., 2021; Draaisma et al., 2022). Therefore, we conclude that, next to the heterogeneity of our target group and high variance of our data, low intensity and frequency mismatch might be possible reasons for our stimulation to show no effect on the level of alpha power, which increased from PRE to POST but irrespective of group.

Our tACS montage has been shown to successfully modify alpha power (Kasten et al., 2016; Stecher et al., 2017) or behavior (Löffler et al., 2018) at the visual cortex. The conventional tACS electrodes used in these studies covered the areas targeted by the stimulation and do not allow EEG recordings; therefore, parietal EEG electrodes were used to estimate occipital alpha power. These were positioned on the spots where the simulation shows maximal field strength. A more focal stimulation with high-definition tACS may be more beneficial for entraining alpha through gamma stimulation—as shown by Helfrich et al. (2014a).

Due to the high technical and organizational expenditure source localization and individual MRI scans were not included in this study. These methods allow the stimulation setup to be adapted to the individual characteristics of each participant. Due to the heterogeneity of the older population, we strongly encourage further tACS research addressing older adults to use individual stimulation setups.

Finally, we can also not exclude the possibility that our EEG analysis masked potential findings. We instructed participants to keep their eyes open during the recordings of the rest EEG but had no possibility of visually checking if participants followed this instruction. Because alpha power changes fundamentally in an eyes-closed or eyes-open condition (Barry and de Blasio, 2017), this is a limitation of the study. Due to eye blinks and other muscular artifacts, data chunks needed to be removed that might have contained relevant information.

Due to difficulties in recruiting participants, we included (as already discussed) participants on medication (which is, in general, also an exclusion criterion in tACS studies) as well as three participants with visual impairment in one eye. Reaction times, amount of errors, and alpha power values were in line with the non-impaired participants. Still, visual impairment (especially monocular vision) may affect vigilance. Monocular vision leads to reduced sensory input, which may lead to increased cognitive load (Casson and Racette, 2000). Furthermore, the field of view is limited, and binocular summation for stereoscopic vision is not possible (Cattaneo et al., 2008). Research has shown that people with visual impairment use compensatory strategies on a behavioral and a neural level (Steeves et al., 2008; Polat et al., 2012). As we used a stimulus positioned centrally in the visual field and tested change of performances on an individual basis, we justified integrating these participants into the overall analysis. Nevertheless, one has to keep in mind that visual impairment might be another influential factor.

### 4.4 Outlook

Recent research has reported on the success of tACS being a promising tool in therapeutic and neuro-enhancement contexts with great potential, especially for older adults (Reinhart and Nguyen, 2019).

Our study suggests that in tACS experiments addressing older adults more factors need to be controlled or participants be measured. It also indicates that many factors, for example, medication, cognitive fitness, education, and digital media, use should be kept in mind. The combination of medication and tACS for treatment success could be beneficial and should be further researched. Paradigms working in young adults should be adjusted and tested in multiple configurations. Higher intensities might be especially profitable. Where possible, individual anatomical and neurophysiological properties should be considered and stimulation frequency mismatch reduced. The altered mechanisms of CFC in aging should be investigated as well.

In general, we recommend considering a*ge* as an important factor and investigating middle-aged cohorts: More studies comparing different age groups are needed to utilize this promising technique for everyday use.

## 5 Conclusion

In this study, we tried to repeat a successful paradigm and tACS setup with a target group aged 65 years and older. We succeeded in inducing a vigilance decrement (rising reaction times with time on task and higher POST alpha power) but could not detect any effect of the intervention with 40-Hz tACS. We conclude that it is not appropriate to simply transfer a successful tACS protocol to an older target group without adjusting stimulation parameters. We observed high variations in all data obtained (reaction times, IAF, and alpha power) that could have masked any effect of intervention. Therefore, we recommend reducing the heterogeneity of the target group for future studies. Next to this, we cannot not exclude the possibility, that older adults differ in their neuro-anatomical characteristics and functioning from a young target group and that our protocol might not be useful for them. Reasons could be the different anatomical and neurophysiological properties of an aged brain, cognitive compensatory mechanisms and the usage of anterior and frontal brain regions or different mindsets and motivations. Therefore, it is necessary to continue research—in the hope that, in the future, not only our bodies will stay healthy and live long but also our minds.

## Data availability statement

The datasets presented in this article are not readily available because datasets are protected by the data privacy concept of the study. They are available by request to the corresponding or BL. Requests to access the datasets should be directed to birte.loeffler@uni-oldenburg.de.

## Ethics statement

The studies involving humans were approved by Medizinische Ethikkommission of the University of Oldenburg. The studies were conducted in accordance with the local legislation and institutional requirements. The participants provided their written informed consent to participate in this study.

## Author contributions

BL, HS, SF, AH, and CH conceptualized the study. BL and AM acquired participants. BL, HS, and AM performed measurements and analyzed the data. BL wrote the manuscript. CH and HS commented and gave practical hints throughout the study. All authors contributed to the article and approved the submitted version.
