# Supplementary material for: Attempting to counteract vigilance decrement in older adults with brain stimulation
Source: Front Neuroergon. 2023 Dec 12;4:1201702. doi: 10.3389/fnrgo.2023.1201702 (PMC10790873; doi:10.3389/fnrgo.2023.1201702)
Supplement: Supplementary file 1 [file Data_Sheet_1.docx]

Supplementary Material

Attempting to Counteract Vigilance Decrement in Older Adults with Brain Stimulation

Birte S. Löffler, Heiko I. Stecher, Arnd Meiser, Sebastian Fudickar, Andreas Hein* & Christoph S. Herrmann

*** Correspondence:** Andreas Hein: Andreas.Hein@uni-oldenburg.de

# Supplementary Tables

## Reaction times analysis:

### LMM: 5 Hz Base

Supplementary Table 1. 5 Hz as base-group. LMM results for reaction times (n = 45, observations = 8413): β presents the regression coefficients, SE β the standard error of β. β_0_ represents the initial reaction time and β_6_ the increase in reaction time over time of the 5 Hz_base_-group (bold font). Their p-values, indexed with a °, show a significant difference compared to zero. All other coefficients are tested against β_0_ and β_6_. Intercepts are given in ms, slopes in ms/min. Model parameters are: R² marginal = 0.056, R² conditional = 0.470 and AIC -9055.448

|  | **β** | **SE beta** | **t-value** | **p-value** |  |  | |
| --- | --- | --- | --- | --- | --- | --- | --- |
| **Intercepts:** |  |  |  |  |  |  | |
| **β_0_ 5 Hz_base_** | **511.7** | **28.0** | **18.29** | **0.000** |  | **< 0.001°** |  |
| β_1_ SHAM_base_ | 58.2 | 40.3 | 1.45 | 0.149 |  | > 0.05 |  |
| β_2_ 40Hz_base_ | 65.4 | 38.9 | 1.68 | 0.093 |  | > 0.05 |  |
| β_3_ 5 Hz_inter_ | 39.5 | 11.6 | 3.40 | 0.001 |  | < 0.01 |  |
| β_4_ SHAM_inter_ | 125.8 | 40.2 | 3.13 | 0.002 |  | < 0.01 |  |
| β_5_ 40Hz_inter_ | 95.8 | 38.9 | 2.46 | 0.014 |  | < 0.05 |  |
|  |  |  |  |  |  |  |  |
| **Slopes:** |  |  |  |  |  |  |  |
| **β_6_ time x 5 Hz_base_** | **1.662** | **0.679** | **2.44** | **0.015** |  | **< 0.05°** |  |
| β_7_ time x SHAM_base_ | 1.746 | 0.974 | 1.79 | 0.073 |  | > 0.05 |  |
| β_8_ time x 40Hz_base_ | 0.420 | 0.941 | 0.45 | 0.654 |  | > 0.05 |  |
| β_9_ time x 5 Hz_inter_ | -0.702 | 0.683 | -1.03 | 0.303 |  | > 0.05 |  |
| β_10_ time x SHAM_inter_ | -0.678 | 0.968 | -0.70 | 0.483 |  | > 0.05 |  |
| β_11_ time x 40Hz_inter_ | 0.936 | 0.937 | 1.00 | 0.317 |  | > 0.05 |  |

### LMM: 40 Hz Base

Supplementary Table 2. 40 Hz as base-group. LMM results for reaction times (n = 45, observations = 8413): β presents the regression coefficients, SE β the standard error of β. β_0_ represents the initial reaction time and β_6_ the increase in reaction time over time of the 40 Hz_base_-group (bold font). Their p-values, indexed with a °, show a significant difference compared to zero. All other coefficients are tested against β_0_ and β_6_. Intercepts are given in ms, slopes in ms/min. Model parameters are: R² marginal = 0.056, R² conditional = 0.470 and AIC -9055.448

|  | **β** | **SE beta** | **t-value** | **p-value** |  |  | |
| --- | --- | --- | --- | --- | --- | --- | --- |
| **Intercepts:** |  |  |  |  |  |  | |
| **β_0_ 40 Hz_base_** | **577.1** | **27.0** | **21.34** | **0.000** |  | **< 0.001°** |  |
| β_1_ 5 Hz_base_ | -65.4 | 38.9 | -1.68 | 0.093 |  | > 0.05 |  |
| β_2_ SHAM_base_ | -7.3 | 39.6 | -0.18 | 0.855 |  | > 0.05 |  |
| β_3_ 40 Hz_inter_ | 30.4 | 11.1 | 2.73 | 0.006 |  | < 0.01 |  |
| β_4_ 5 Hz_inter_ | -26.0 | 38.8 | -0.67 | 0.504 |  | > 0.05 |  |
| β_5_ SHAM_inter_ | 60.4 | 39.5 | 1.53 | 0.127 |  | > 0.05 |  |
|  |  |  |  |  |  |  |  |
| **Slopes:** |  |  |  |  |  |  |  |
| **β_6_ time x 40 Hz_base_** | **2.082** | **0.652** | **3.19** | **0.001** |  | **< 0.01°** |  |
| β_7_ time x 5 Hz_base_ | -0.420 | 0.941 | -0.45 | 0.654 |  | > 0.05 |  |
| β_8_ time x SHAM_base_ | 1.320 | 0.955 | 1.38 | 0.166 |  | > 0.05 |  |
| β_9_ time x 40 Hz_inter_ | 0.516 | 0.655 | 0.79 | 0.432 |  | > 0.05 |  |
| β_10_ time x 5 Hz_inter_ | -1.128 | 0.932 | -1.21 | 0.227 |  | > 0.05 |  |
| β_11_ time x SHAM_inter_ | -1.104 | 0.949 | -1.16 | 0.246 |  | > 0.05 |  |

### LMM: Factor *medication*

Supplementary Table 3. LMM results for reaction times (n = 45, observations = 8413) with cardiovascular medication (yes/no) as the only factor: β presents the regression coefficients, SE β the standard error of β. β_0_ represents the initial reaction time and β_2_ the increase in reaction time over time for the healthy, i.e. participants without medication (bold font). Their p-values, indexed with a °, show a significant difference compared to zero. The other coefficients are tested against β_0_ and β_2_. Intercepts are given in ms, slopes in ms/min. Model parameters are: R² marginal = 0.021, R² conditional = 0.467 and AIC -9076.73

|  | **β** | **SE beta** | **t-value** | **p-value** |  |  | |
| --- | --- | --- | --- | --- | --- | --- | --- |
| **Intercepts:** |  |  |  |  |  |  | |
| **β_0_ healthy** | **586.4** | **24.4** | **24.04** | **0.000°** |  | **< 0.001°** |  |
| β_1_ medication | -18.4 | 32.7 | -0.56 | 0.576 |  | > 0.05 |  |
|  |  |  |  |  |  |  |  |
| **Slopes:** |  |  |  |  |  |  |  |
| **β_2_ time x healthy** | **2.796** | **0.476** | **5.87** | **0.000°** |  | **< 0.001°** |  |
| β_3_ time x medication | -1.584 | 0.638 | -2.48 | 0.013 |  | < 0.05 |  |

Supplementary Table 4. LMM results for reaction times (n = 45, observations = 8413) with cardiovascular medication (yes/no), *group*, and their interaction over time as factors: β presents the regression coefficients, SE β the standard error of β. β_0_ represents the initial reaction time and β_6_ the increase in reaction time over time of the SHAM_base_-group without medication (bold font). Their p-values, indexed with a °, show a significant difference compared to zero. All other coefficients are tested against β_0_ and β_6_. Intercepts are given in ms, slopes in ms/min. Model parameters are: R² marginal = 0.152, R² conditional = 0.462 and AIC -8959.546

|  | **β** | **SE beta** | **t-value** | **p-value** |  |  | |
| --- | --- | --- | --- | --- | --- | --- | --- |
| **Intercepts:** |  |  |  |  |  |  | |
| **β_0_ SHAM_base_** | **495.0** | **41.7** | **11.86** | **0.000** |  | **< 0.001°** |  |
| β_1_ 5Hz_base_ | 41.6 | 55.2 | 0.75 | 0.451 |  | > 0.05 |  |
| β_2_ 40Hz_base_ | 122.8 | 589.8 | 2.08 | 0.037 |  | < 0.05 |  |
| β_3_ SHAM_inter_ | 103.3 | 18.1 | 5.72 | 0.000 |  | < 0.001 |  |
| β_4_ 5Hz_inter_ | 103.2 | 55.1 | 1.87 | 0.612 |  | > 0.05 |  |
| β_5_ 40Hz_inter_ | 181.4 | 58.9 | 3.08 | 0.002 |  | < 0.01 |  |
| β_6_ SHAM_base_ + medication | 130.2 | 55.1 | 2.36 | 0.229 |  | > 0.05 |  |
| β_7_ 5Hz_base_ + medication | -183.5 | 76.5 | -2.40 | 0.016 |  | < 0.05 |  |
| β_8_ 40Hz_base_ + medication | -195.4 | 76.3 | -2.56 | 0.011 |  | < 0.05 |  |
| β_9_ SHAM_inter_ + medication | -61.7 | 23.8 | -2.59 | 0.010 |  | < 0.05 |  |
| β_10_ 5Hz_inter_ + medication | -230.9 | 76.3 | -3.03 | 0.003 |  | < 0.01 |  |
| β_11_ 40Hz_inter_ + medication | -240.5 | 76.2 | -3.16 | 0.002 |  | < 0.01 |  |
|  |  |  |  |  |  |  |  |
| **Slopes:** |  |  |  |  |  |  |  |
| **β_12_ time x SHAM_base_** | **3.624** | **0.993** | **3.65** | **0.000** |  | **< 0.001°** |  |
| β_13_ time x 5Hz_base_ | -1.05 | 1.315 | -0.80 | 0.425 |  | > 0.05 |  |
| β_14_ time x 40Hz_base_ | 0.264 | 1.400 | 0.19 | 0.850 |  | > 0.05 |  |
| β_15_ time x SHAM_inter_ | -2.376 | 1.059 | -2.24 | 0.025 |  | < 0.05 |  |
| β_16_ time x 5Hz_inter_ | -2.28 | 1.305 | -1.75 | 0.081 |  | > 0.05 |  |
| β_17_ time x 40Hz_inter_ | 1.404 | 1.389 | 1.01 | 0.313 |  | > 0.05 |  |
| β_18_ time x SHAM_base_ x medication | -0.354 | 1.308 | -0.27 | 0.788 |  | > 0.05 |  |
| β_19_ time x 5Hz_base_ x medication | -1.62 | 1.819 | -0.89 | 0.374 |  | > 0.05 |  |
| β_20_ time x 40Hz_base_ x medication | -2.538 | 1.808 | -1.40 | 0.161 |  | > 0.05 |  |
| β_21_ time x SHAM_inter_ x medication | -0.132 | 1.399 | -0.09 | 0.926 |  | > 0.05 |  |
| β_22_ time x 5Hz_inter_ x medication | -0.486 | 1.800 | -0.27 | 0.788 |  | > 0.05 |  |
| β_23_ time x 40Hz_inter_ x medication | -3.552 | 1.796 | -1.98 | 0.048 |  | < 0.05 |  |
